# Structure of heme *d*_1_-free *cd*_1_ nitrite reductase NirS

**DOI:** 10.1107/S2053230X20006676

**Published:** 2020-05-29

**Authors:** Thomas Klünemann, Wulf Blankenfeldt

**Affiliations:** a Structure and Function of Proteins, Helmholtz Centre for Infection Research, Inhoffenstrasse 7, 38124 Braunschweig, Niedersachsen, Germany; b Institute for Biochemistry, Biotechnology and Bioinformatics, Technische Universität Braunschweig, Spielmannstrasse 7, 38106 Braunschweig, Niedersachsen, Germany

**Keywords:** *cd*_1_ nitrite reductase, NirS, heme *d*_1_

## Abstract

A key step in anaerobic nitrate respiration is the reduction of nitrite to nitric oxide, which is catalysed by the *cd*_1_ nitrite reductase NirS in, for example, the Gram-negative opportunistic pathogen *Pseudomonas aeruginosa*. Each subunit of this homodimeric enzyme consists of a cytochrome *c* domain and an eight-bladed β-propeller that binds the uncommon isobacteriochlorin heme *d*_1_ as an essential part of its active site. Although NirS has been well studied mechanistically and structurally, the focus of previous studies has been on the active heme *d*_1_-bound form. The heme *d*_1_-free form of NirS reported here, which represents a premature state of the reductase, adopts an open conformation with the cytochrome *c* domains moved away from each other with respect to the active enzyme. Further, the movement of a loop around Trp498 seems to be related to a widening of the propeller, allowing easier access to the heme *d*_1_-binding side. Finally, a possible link between the open conformation of NirS and flagella formation in *P. aeruginosa* is discussed.

## Abstract 2

The crystal structure of a heme *d*_1_-free form of the *cd*_1_ nitrite reductase NirS from *Pseudomonas aeruginosa* has been determined and provides insight into a premature form of the enzyme.

## 1. Introduction   

**Figure 1:**
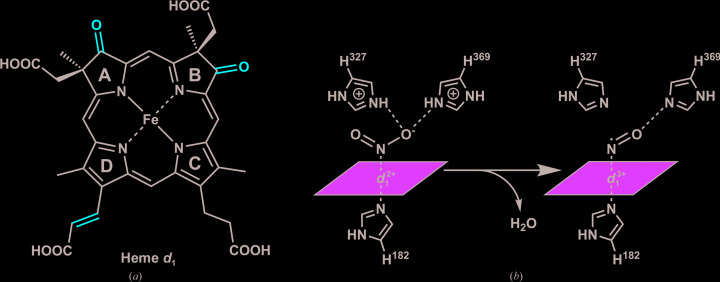
(*a*) Chemical structure of heme *d*_1_ with characteristic features highlighted in red. (*b*) Simplified depiction of the *Pa*-NirS active site during the reduction of nitrite to nitric oxide.

Denitrification is the stepwise reduction of nitrogen oxides to dinitrogen and is utilized by many bacteria to replace the terminal oxygen-dependent steps of the respiratory chain under anaerobic conditions. The reduction of nitrite to nitric oxide is a highly regulated step, since both of these molecules are toxic to the cell. Many bacteria utilize the *cd*_1_ nitrite reductase NirS to catalyse this step, and the NirS enzymes from *Pseudomonas aeruginosa* (*Pa*-NirS) and *Paracoccus pantotrophus* (*Pp*-NirS) have been well studied functionally and structurally. Both chains of this homodimeric enzyme possess a *c*-type cytochrome domain with a covalently attached heme *c* functioning as an electron-entry point and a C-terminal eight-bladed β-propeller that utilizes the uncommon iso­bacteriochlorin heme *d*_1_ as an active-site cofactor (Fig. 1 ▸ *a*; Nurizzo *et al.*, 1997 ▸; Williams *et al.*, 1997 ▸). The domains interact via an N-terminal arm which, in the case of *Pa*-NirS, belongs to the opposing subunit of the homodimer (Williams *et al.*, 1997 ▸; Nurizzo *et al.*, 1997 ▸). Compared with other tetrapyrroles such as heme *b*, a higher affinity for anionic molecules such as nitrite and a lower affinity for nitric oxide has been observed for the ferrous isobacteriochlorin heme *d*_1_. This is rooted in the unique carbonyl moieties at rings A and B and the replacement of the typical propionate by an acrylate at ring D (Chang *et al.*, 1986 ▸; Rinaldo *et al.*, 2011 ▸; Fujii *et al.*, 2016 ▸). During its reduction, nitrite is coordinated by His327 and His369 on top of the ferrous iron of heme *d*_1_ (Fig. 1 ▸ *b*; Cutruzzolà *et al.*, 2001 ▸). After dehydration and reduction of the substrate, the tetrapyrrole heme *d*_1_ is then reduced via internal electron transfer to allow efficient replacement of the product nitric oxide by another nitrite anion (Rinaldo *et al.*, 2011 ▸). In the case of *Pa*-NirS, the intramolecular electron transfer is the rate-limiting step and is allosterically controlled by smaller conformational changes within the cytochrome *c* domain (Farver *et al.*, 2009 ▸; Nurizzo *et al.*, 1999 ▸, 1998 ▸). Interestingly, when *Pp*-NirS was crystallized under reducing conditions, an ∼60° rotation of the cytochrome *c* domain around the pseudo-eightfold axis of the β-propeller was observed (Sjögren & Hajdu, 2001 ▸). This rotation was also seen in *Pa*-NirS after the mutation of His327 to alanine (Brown *et al.*, 2001 ▸).

All structural studies of NirS to date have focused on the heme *d*_1_-bound form of the enzyme and have led to a detailed understanding of the reaction mechanism within the context of denitrification. Recently, however, following the discovery that *Pa*-NirS forms complexes with the flagella protein FliC and the chaperone DnaK, an enzyme activity-independent scaffolding function of a heme *d*_1_-free form of NirS in flagella formation has been proposed (Borrero-de Acuña *et al.*, 2015 ▸). This cofactor-free form is also expected to exist in the course of maturation of NirS, where the enzyme has been demonstrated to transiently interact with NirF and NirN, two proteins that are involved in the biosynthesis of the heme *d*_1_ cofactor (Nicke *et al.*, 2013 ▸). We have therefore set out to determine the heme *d*_1_-free structure of NirS in order to gain more insight into these processes.

## 2. Materials and methods   

### 2.1. Macromolecule production   

NirS with bound dihydro-heme *d*_1_ was isolated from *P. aeruginosa* strain RM361 (*nirN::tet*) (Kawasaki *et al.*, 1997 ▸) after cultivation under anaerobic conditions as described previously (Adamczack *et al.*, 2014 ▸; Klünemann *et al.*, 2019 ▸). Briefly, purification of NirS with bound dihydroheme *d*_1_, the precursor of heme *d*_1_ that accumulates in the absence of NirN, was achieved by two subsequent ion-exchange chromatographic steps, as first described by Parr *et al.* (1976 ▸). The purified protein was dialysed against 10 m*M* Tris–HCl pH 8 and loaded onto a Q-Sepharose Fast Flow anion-exchange column. After washing for 4 h with dialysis buffer at a flow rate of 1 ml min^−1^ the eluted protein lost dihydro-heme *d*_1_, but still contained the covalently attached heme *c*, as indicated by a UV–Vis absorption band at 410 nm (indicative of heme *c*) and no absorption at 630 nm (indicative of dihydro-heme *d*_1_) during elution. Interestingly, dihydro-heme *d*_1_ did not seem to be eluted during the washing procedure but remained bound to the resin, and was visible as a greenish coloration of the chromatography column. Prior to crystallization, the protein was subjected to size-exclusion chromatography (SEC; Superdex 200, GE Healthcare) in a buffer consisting of 10 m*M* Tris–HCl pH 8, 150 m*M* NaCl.

### 2.2. Crystallization   

**Figure 2:**
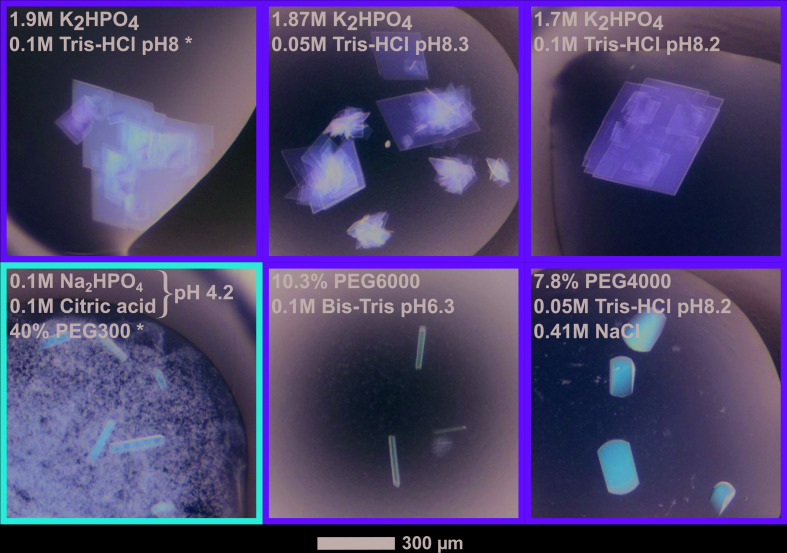
Photographs of representative crystals grown from NirS isolated with (green frame) or without (red frame) bound dihydro-heme *d*_1_. The data sets used in this study were collected from the crystallization conditions marked with an asterisk (*).

Crystallization was performed by sitting-drop vapour diffusion in Intelli-Plates 96-3 (Art Robbins Instruments) at room temperature. Plates were set up using a HoneyBee 961 pipetting robot (Digilab Genomic Solutions), which mixed 200 nl protein solution with 200 nl precipitant solution and provided a reservoir of 60 µl. To obtain structural insight into possible changes in the dihydro-heme *d*_1_-bound form, a screen based on previously published crystallization conditions for NirS (Tegoni *et al.*, 1994 ▸; Brown *et al.*, 2001 ▸) was performed. After two days, plate-shaped green crystals were observed with phosphate as the precipitant (0.1 *M* Tris–HCl pH 8, 1.9 *M* K_2_HPO_4_). The green colour hinted at the presence of dihydro-heme *d*_1_ in these crystals (Fig. 2 ▸). Surprisingly, under conditions containing PEG 4000 or PEG 6000, red rod-shaped or tetragonal crystals started to grow. The red coloration indicated that dihydro-heme *d*_1_ had been lost as a cofactor. As these crystals diffracted X-rays to only 4 Å resolution, sparse-matrix screening with the commercially available crystallization suites JCSG+ (Qiagen) and Morpheus (Molecular Dimensions) was used to identify conditions that were better suited to produce crystals for diffraction experiments. The best-diffracting crystals of heme *d*_1_-free NirS were obtained with 0.1 *M* disodium hydrogen phosphate, 0.1 *M* citric acid, 40%(*v*/*v*) PEG 300 at pH 4.2 using NirS with dihydro-heme *d*_1_ removed by the chromatographic procedure described above prior to the crystallization experiment. Crystals were flash-cooled in liquid nitrogen after cryoprotection with 10%(*v*/*v*) (*R*,*R*)-2,3-butanediol.

### 2.3. Data collection and processing   

For both data sets, 3600 diffraction images, each with an oscillation angle of 0.1°, were collected on beamline P11 at PETRA III, DESY, Hamburg, Germany with a PILATUS 6M fast detector. Images were processed utilizing the *autoPROC* pipeline (Vonrhein *et al.*, 2011 ▸) executing *XDS* (Kabsch, 2010 ▸), *POINTLESS* (Evans, 2011 ▸) and *AIMLESS* (Evans & Murshudov, 2013 ▸). In the case of NirS with bound dihydro-heme *d*_1_ the crystals diffracted anisotropically, which was accounted for using *STARANISO* (Tickle *et al.*, 2018 ▸) within *autoPROC*. Data-collection and processing statistics are summarized in Table 1 ▸.

### 2.4. Structure solution and refinement   

The crystal structure of heme *d*_1_-free NirS was determined by molecular replacement utilizing *Phaser* (McCoy *et al.*, 2007 ▸) and the coordinates of the H327A variant of *Pa*-NirS (NirS^H327A^; PDB entry 1hzu; Brown *et al.*, 2001 ▸), which crystallized in the same space group but with unit-cell axes that were approximately 3 Å longer. The structure of NirS with bound dihydro-heme *d*_1_ was phased by Fourier synthesis using a published structure of *Pa*-NirS with bound heme *d*_1_ as a starting model (PDB entry 1nir; Nurizzo *et al.*, 1997 ▸). To remove phase bias, both initial models were subjected to ten cycles of refinement with simulated annealing enabled in *phenix.refine* (Afonine *et al.*, 2012 ▸). The final models were constructed by several cycles of manual optimization in *Coot* (Emsley *et al.*, 2010 ▸) and computational refinement including TLS refinement. Depictions of the final models were prepared with *PyMOL* (version 1.8; Schrödinger). Refinement statistics are summarized in Table 2 ▸.

## 3. Results   

**Figure 3:**
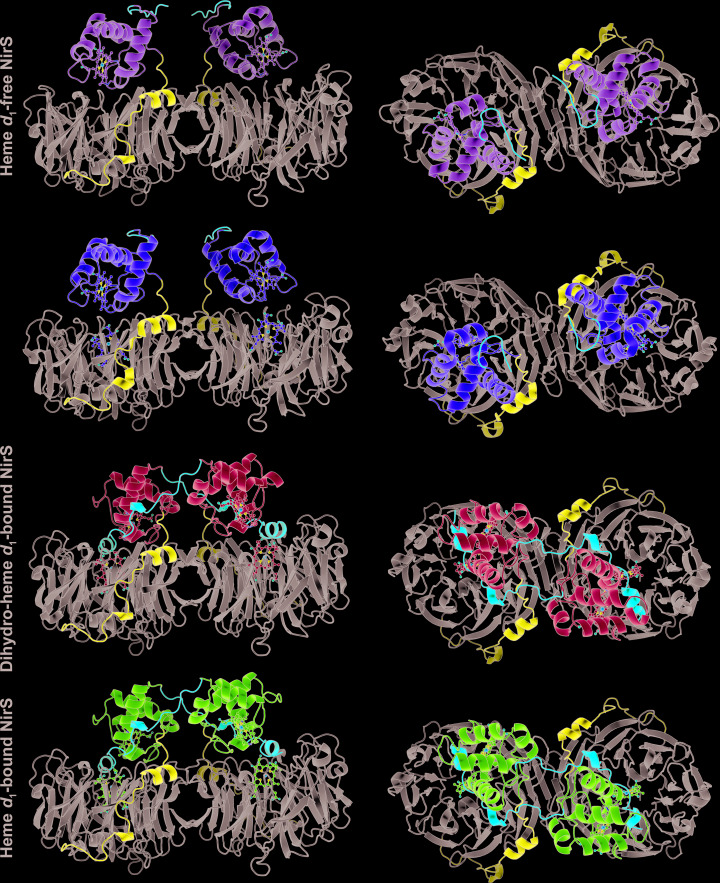
Cartoon representations of the different conformations observed for the *cd*_1_ nitrite reductase NirS from *P. aeruginosa* (heme *d*_1_-bound NirS, PDB entry 1nir, Nurizzo *et al.*, 1997 ▸; NirS^H327A^, PDB entry 1hzu, Brown *et al.*, 2001 ▸) after superposition of the *d*_1_ domains. The N-terminal arm is coloured red, the linker blue and the *d*_1_ domain grey. The cytochrome *c* domain and the tetrapyrroles are coloured individually.

In previous work with the dihydro-heme *d*_1_ dehydrogenase NirN, we isolated the *cd*_1_ nitrite reductase NirS with bound dihydro-heme *d*_1_, the final intermediate of heme *d*_1_ biosynthesis, which differs from heme *d*_1_ by lacking a double bond in the propionate chain attached to ring D (Klünemann *et al.*, 2019 ▸). It has previously been shown that NirS can utilize both cofactors but is less active with dihydro-heme *d*_1_ (Hasegawa *et al.*, 2001 ▸; Kawasaki *et al.*, 1997 ▸; Adamczack *et al.*, 2014 ▸). To determine whether the altered activity is caused by structural changes or by the inherent properties of dihydro-heme *d*_1_, NirS with bound dihydro-heme *d*_1_ was crystallized using the published conditions for heme *d*_1_-bound NirS (Tegoni *et al.*, 1994 ▸; Brown *et al.*, 2001 ▸). In this complex, anomalous difference density around the iron centres indicates similar occupancies for dihydro-heme *d*_1_ and heme *c*, with the latter being covalently attached to the polypeptide. Comparison with the previously published heme *d*_1_-bound structure of NirS reveals no significant differences (Nurizzo *et al.*, 1997 ▸; C^α^ r.m.s.d. of 0.38 Å; Figs. 3 ▸ and 4 ▸). This suggests that the reported differences in the activity of NirS loaded with dihydro-heme *d*_1_ or heme *d*_1_ are likely to be caused by inherent properties of the cofactor.

The structure of heme *d*_1_-free NirS possesses a similar dimeric structure and domain organization as mature heme *d*_1_-bound NirS (Nurizzo *et al.*, 1997 ▸). Both chains have a C-terminal eight-bladed β-propeller, which has previously been termed the *d*_1_ domain as it is responsible for binding *d*_1_-type tetrapyrroles in NirS and the related proteins NirF and NirN (Fig. 3 ▸). In NirS, these domains form homodimers by interaction of the 15th β-strand of each subunit, which is consistent with the SEC data collected during purification (data not shown). The N-terminal domain consists of a cytochrome *c* fold that is connected *via* a linker domain to the *d*_1_ domain and harbours a covalently attached heme *c*. Interestingly, the position of the cytochrome *c* domain differs from that found in the heme *d*_1_-bound and dihydro-heme *d*_1_-bound structures by a rotation of approximately 60° around the pseudo-eightfold axis of the β-propeller. A similar conformation has previously been observed for *Pa*-NirS after amino-acid exchange of His327 to alanine or for *Pp*-NirS crystallized under reducing conditions (Brown *et al.*, 2001 ▸; Sjögren & Hajdu, 2001 ▸). In both of these structures, as well as in the structure of heme *d*_1_-free NirS reported here, no density is observed for the N-terminal arm, which resides between the cytochrome *c* domain and the *d*_1_ domain of the opposing subunit in *Pa*-NirS in the closed conformation, indicating that it is flexible in the open conformation.

**Figure 4:**
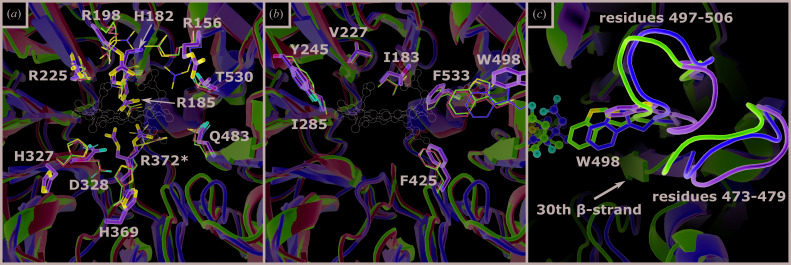
Depiction of hydrophilic (*a*) and hydrophobic (*b*) residues in the heme *d*_1_-binding pocket. Amino acids are shown as sticks (heme *d*_1_-free NirS, green) or lines (heme *d*_1_-bound NirS, magenta; NirS^H327A^, yellow; dihydro-heme *d*_1_-bound NirS, cyan). Residues with alternative conformations are marked with an asterisk (*). The position of heme *d*_1_ inside the binding pocket is shown as a thin outline. (*c*) shows the movement of the loops associated with Trp498 narrowing the binding pocket of heme *d*_1_-free NirS after binding a *d*_1_-type heme

An overlay of the cytochrome *c* domain of heme *d*_1_-free NirS with other *Pa*-NirS structures reveals a higher similarity to the structure of NirS reduced *in crystallo* (C^α^ r.m.s.d. of 0.37 Å) compared with the oxidized form (C^α^ r.m.s.d. of 1.09 Å) (Nurizzo *et al.*, 1997 ▸, 1998 ▸). The difference is mostly caused by the position of residues 55–64 and is consistent with an observation previously discussed for NirS^H327A^ (C^α^ r.m.s.d. for cytochrome *c* of 0.35 Å). A similar comparison of the *d*_1_ domain reveals a lower C^α^ r.m.s.d. with respect to the H327A variant of *Pa*-NirS (0.75 Å) than to the oxidized or reduced wild-type protein (1.34 and 1.39 Å, respectively). NirS^H327A^ has been shown to have a wider β-propeller, allowing better solvent access to heme *d*_1_ (Brown *et al.*, 2001 ▸). Furthermore, the reported occupancy (0.5) of heme *d*_1_, together with the observed change in coloration of these crystals over time, indicating the loss of heme *d*_1_, suggests that the open conformation allows easy access to the ligand-binding site in general (Brown *et al.*, 2001 ▸). Closer inspection of the heme *d*_1_-binding pocket reveals only a few distinct changes related to *d*_1_-type heme binding (Fig. 4 ▸). Three arginines that interact with the acetate or propionate side chains of heme *d*_1_ in the heme *d*_1_-bound structure (Arg156, Arg198 and Arg372) adopt different conformations in the heme *d*_1_-free form. Additionally, His327 and His369, which are both involved in substrate binding and catalysis by NirS, have different positions. Interestingly, His182, which acts as the fifth ligand of the iron cation in the centre of heme *d*_1_, shows no conformational changes without bound tetrapyrrole. This stands in contrast to the *d*_1_-type heme-binding β-propellers of the heme *d*_1_-biosynthesis proteins NirN and NirF, in which the corresponding histidine rotates with respect to the ligand-free form to coordinate the iron (Klünemann *et al.*, 2020 ▸, 2019 ▸). Another distinctive variation in the *d*_1_ domains of heme *d*_1_-free and heme *d*_1_-bound NirS is observed for the loop consisting of residues 497–506, which moves upon heme *d*_1_ binding to enable Trp498 to lock the cofactor inside its binding pocket. Interestingly, the neighbouring loop (residues 473–479) also adopts a slightly different conformation. This loop is connected to the 30th β-strand, which is inside the propeller and moves inwards upon heme *d*_1_ binding, indicating a connection between the widening of the propeller and the movement of Trp498 (Fig. 4 ▸ *c*).

## 4. Discussion   

It has long been known that the *cd*_1_ nitrite reductase NirS adopts an open and a closed conformation, and it has been hypothesized that these states are linked to the catalytic cycle (Brown *et al.*, 2001 ▸; Sjögren & Hajdu, 2001 ▸). The data presented here extend the potential importance of the open conformation by suggesting that it also represents a native but immature form of the enzyme that facilitates binding of the heme *d*_1_ cofactor as the ultimate step of NirS maturation.

The open conformation of NirS may also have additional physiological importance outside the denitrification process. It has recently been found that NirS can function as scaffold protein for flagella formation under denitrifying conditions, even in the absence of heme *d*_1_ (Borrero-de Acuña *et al.*, 2015 ▸). Interestingly, the N-terminal arm and the cytochrome *c* domain of NirS have been shown to be involved in complex formation between NirS, FliC and DnaK in this process (Borrero-de Acuña *et al.*, 2015 ▸). Further, kinetic studies with NirS from *Pseudomonas stutzeri*, which lacks the N-terminal arm, and the mutation of a conserved tyrosine in *Pa*-NirS and *Pp*-NirS suggest that the N-terminal arm is not required for the enzymatic activity, despite its interaction with heme *d*_1_ (Cutruzzolà *et al.*, 1997 ▸; Gordon *et al.*, 2003 ▸; Wilson *et al.*, 2001 ▸). Therefore, the conformational change observed in our study can be envisioned to release the N-terminal arm to allow flagella formation, thereby regulating cell motility. Further investigation will be required to confirm that the conformational changes observed *in crystallo* can also be found in solution and are associated with distinct functions of NirS *in vivo*.

## Supplementary Material

PDB reference: NirS, without bound heme *d*_1_, 6tpo

PDB reference: with bound dihydro-heme *d*_1_, 6tsi

## Figures and Tables

**Table 1:** Data collection and processing Values in parentheses are for the outer shell.

	Heme *d*_1_-free NirS	Dihydro-heme *d*_1_-bound NirS
Diffraction source	P11, PETRA III	P11, PETRA III
Wavelength (Å)	1.033	1.739
Temperature (K)	100	100
Detector	PILATUS 6M fast	PILATUS 6M fast
Crystal-to-detector distance (mm)	368.1	168.4
Rotation range per image (°)	0.1	0.1
Total rotation range (°)	360	360
Exposure time per image (s)	0.1	0.05
Space group	*P*4_3_22	*P*2_1_2_1_2
*a*, *b*, *c* (Å)	67.25, 67.25, 277.10	163.76, 91.09, 112.71
α, β, γ (°)	90, 90, 90	90, 90, 90
Resolution range (Å)	48.25–1.86 (1.93–1.86)	112.71–2.38 (2.62–2.38)
Resolution limits along axes (a*, b*, c*)	—	2.89, 2.38, 2.51
Total No. of reflections	1363122 (93274)	625785 (23996)
No. of unique reflections	54709 (5373)	50095 (2505)
Completeness, spherical (%)	99.97 (100)	73.1 (14.7)
Completeness, ellipsoidal (%)	—	92.6 (44.5)
Multiplicity	24.9 (17.4)	12.5 (9.6)
〈*I*/σ(*I*)〉	20.3 (1.8)	6.6 (1.6)
CC_1/2_	0.99 (0.57)	0.99 (0.64)
*R*_p.i.m._	0.03 (0.32)	0.11 (0.54)
Overall *B* factor from Wilson plot (Å^2^)	26.2	23.6

**Table 2:** Structure solution and refinement Values in parentheses are for the outer shell.

	Heme *d*_1_-free NirS	Dihydro-heme *d*_1_-bound NirS
Resolution range (Å)	48.25–1.86 (1.93–1.86)	79.61–2.38 (2.42–2.38)
Final *R*_cryst_	0.17 (0.23)	0.24 (0.33)
Final *R*_free_	0.19 (0.27)	0.28 (0.43)
No. of non-H atoms		
Protein	4100	8364
Ligand	65	214
Water	255	246
R.m.s. deviations		
Bonds (Å)	0.01	0.01
Angles (°)	0.94	1.37
Average *B* factor (Å^2^)	31.0	23.6
Ramachandran plot		
Most favoured (%)	96.35	94.24
Allowed (%)	3.65	5.67
